# Myocardial Injury in Rheumatic Diseases: Immune and Microcirculatory and Molecular Mechanisms of Cardiomyopathies

**DOI:** 10.3390/ijms27125513

**Published:** 2026-06-18

**Authors:** Mateusz Lucki, Ewa Lucka, Bogna Grygiel-Górniak, Sylwia Iwańczyk, Przemysław Mitkowski, Maciej Lesiak

**Affiliations:** 1 Department and Clinic of Cardiology, University of Medical Sciences, 60-545 Poznań, Polandsiwanczyk@ump.edu.pl (S.I.); przemyslaw.mitkowski@ump.edu.pl (P.M.); maciej.lesiak@ump.edu.pl (M.L.); 2 Clinical Rehabilitation Laboratory, Department of Rehabilitation and Physiotherapy, University of Medical Sciences, 60-545 Poznań, Poland; 3 Department of Rheumatology, Rehabilitation and Internal Diseases, Poznan University of Medical Sciences, 61-701 Poznań, Poland; bgrygiel@ump.edu.pl

**Keywords:** autoimmune diseases, myocarditis, rheumatic diseases, microcirculation, vascular endothelium, immunity, innate, cytokines, cardiomyopathies

## Abstract

Immune-mediated myocardial injury is an important yet underrecognized manifestation of systemic rheumatic diseases and represents a biologically heterogeneous process extending beyond traditional cardiovascular complications such as pericardial disease or accelerated atherosclerosis. This review aimed to summarize current evidence regarding the molecular mechanisms underlying autoimmune myocardial injury and propose an integrated pathogenic framework. A structured narrative review of the literature was performed, focusing on molecular and cellular mechanisms, disease-specific pathogenic pathways, advances in cardio-immunology, and contemporary diagnostic approaches in autoimmune myocardial disease. Current evidence indicates that myocardial injury in rheumatic diseases results from complex interactions involving autoantibody-mediated injury, immune complex deposition, endothelial dysfunction, coronary microvascular dysfunction, dysregulated innate and adaptive immunity, oxidative stress, mitochondrial dysfunction, immunometabolic reprogramming, and regulated cardiomyocyte death. These mechanisms contribute to heterogeneous clinical manifestations, including myocarditis, arrhythmias, inflammatory cardiomyopathy, and heart failure. An integrated immune–microvascular–immunometabolic framework may represent a central mechanism driving myocardial injury and progression to inflammatory cardiomyopathy, supporting earlier diagnosis, improved risk stratification, and the development of precision therapeutic strategies.

## 1. Introduction

Cardiovascular disease represents a major determinant of morbidity and mortality among patients with systemic autoimmune and rheumatic disorders. Traditionally, cardiovascular involvement in these conditions has been interpreted through manifestations such as pericarditis [1], valvular abnormalities [2], pulmonary hypertension [3], accelerated atherosclerosis [4], thromboembolic complications [5], and treatment-related cardiotoxicity [6]. However, recent advances in cardiovascular imaging [7,8,9,10], together with emerging evidence of coronary microvascular dysfunction and subclinical myocardial abnormalities in systemic lupus erythematosus and rheumatoid arthritis [11,12,13,14,15,16,17,18,19,20], as well as translational studies investigating inflammatory and immunometabolic pathways [19,20,21,22,23,24,25,26], indicate that direct myocardial involvement is both more prevalent and biologically more complex than previously recognized [1,4]. Recent European Society of Cardiology (ESC) guidelines provide an updated and comprehensive framework for the diagnosis and management of inflammatory myocardial syndromes, emphasizing multimodal imaging, disease phenotyping, and underlying pathophysiological mechanisms [9]. This integrative approach aligns closely with emerging evidence linking immune activation, endothelial dysfunction, coronary microvascular impairment, mitochondrial dysfunction, and immunometabolic reprogramming to myocardial injury and progression toward inflammatory cardiomyopathy [21,22,23,24,25,26,27,28].

Myocardial involvement has been reported in approximately 20–50% of patients with systemic autoimmune diseases depending on the underlying disease entity and diagnostic modality used, with substantially higher prevalence detected using cardiac magnetic resonance imaging and biomarker-based screening [24,25]. In these patients, the myocardium becomes a direct target of immune-mediated injury, manifesting as overt myocarditis or as a more subtle inflammatory myocardial disease that may progress toward inflammatory cardiomyopathy, fibrosis, electrical instability, and ventricular dysfunction [24,25]. At the cellular level, cardiomyocytes, endothelial cells, and cardiac fibroblasts represent interconnected targets of injury, linking immune activation with impaired myocardial energetics, microvascular dysfunction, and maladaptive structural remodeling [29,30].

Importantly, myocardial involvement in systemic autoimmunity rarely reflects a single pathogenic mechanism but arises from the interaction of systemic immune dysregulation [26], endothelial activation [27], coronary microvascular dysfunction (CMD) [29], inflammatory cell infiltration [29], autoantibody-mediated injury [31,32], complement activation [33,34], metabolic stress—including mitochondrial dysfunction, oxidative stress, altered substrate utilization, and immunometabolic reprogramming [21,22,26,35]—and fibro-inflammatory remodeling [30]. Among these, CMD has emerged as a key mechanism translating systemic inflammation into myocardial ischemia and tissue injury [36,37,38,39]. Even in the absence of obstructive epicardial coronary artery disease, microvascular dysfunction may impair myocardial perfusion, promote oxidative stress, and disrupt cardiomyocyte energetics. Clinical studies have demonstrated coronary microvascular abnormalities in systemic lupus erythematosus [34], women with autoimmune rheumatic diseases participating in the WISE-CVD project [35], and patients with systemic inflammatory disorders undergoing advanced functional assessment of coronary microcirculation [28]. These findings support the concept that CMD contributes substantially to inflammatory myocardial injury in systemic autoimmune diseases [34,35]. This mechanistic complexity contributes directly to diagnostic challenges. Clinical manifestations are often nonspecific and include exertional dyspnea [12], fatigue [24], palpitations [25], or chest discomfort [24], while cardiac biomarkers may be only mildly elevated [34,35,39]. Conventional echocardiography frequently fails to detect early or patchy myocardial inflammation, highlighting the importance of advanced cardiac imaging modalities [2,3,9,40]. Cardiac magnetic resonance studies have demonstrated subclinical myocardial abnormalities in systemic lupus erythematosus [41], altered myocardial tissue characteristics detected by T1 and T2 mapping [41], and early myocardial remodeling in rheumatoid arthritis even in the absence of clinically overt cardiovascular disease [15,42]. At the severe end of the spectrum, myocardial involvement may present with acute heart failure, malignant arrhythmias, cardiogenic shock, or sudden cardiac death [12]. More commonly, however, it follows a smoldering course characterized by low-grade inflammation and repeated microvascular or immune-mediated injury leading to progressive structural and functional myocardial alterations [43].

From a pathobiological perspective, myocardial inflammation in systemic rheumatic diseases follows disease-specific patterns. Immune complex–mediated inflammation and type I interferon signaling dominate in systemic lupus erythematosus [13,44]. Advanced imaging studies have demonstrated coronary microvascular dysfunction [28], diffuse myocardial abnormalities detected by native T1/T2 mapping [13], and clinically silent myocardial involvement missed by conventional echocardiography [14]. In idiopathic inflammatory myopathies, myocardial involvement reflects extension of striated muscle autoimmunity [15,17,18,19,20]. Cardiovascular magnetic resonance studies have identified characteristic patterns of myocardial inflammation, edema, and fibrosis, frequently preceding overt cardiac symptoms [45,46].

Rheumatoid arthritis, in contrast, promotes myocardial injury primarily through chronic cytokine-driven inflammation, endothelial dysfunction, accelerated atherosclerosis, coronary microvascular abnormalities, and progressive myocardial remodeling. Clinical studies have demonstrated that cumulative inflammatory burden contributes to the progression of coronary atherosclerosis and cardiovascular risk in patients with rheumatoid arthritis [17]. Furthermore, cardiovascular magnetic resonance imaging has identified early myocardial abnormalities, including altered myocardial tissue characteristics, diffuse myocardial remodeling, and evidence of subclinical myocardial inflammation even in patients without clinically overt cardiovascular disease [15,18].

Positron emission tomography studies have further demonstrated impaired coronary microvascular function in rheumatoid arthritis, supporting the contribution of coronary microvascular dysfunction to myocardial injury and adverse cardiovascular outcomes [47,48]. Moreover, cardiovascular magnetic resonance investigations have demonstrated diffuse myocardial fibrosis and extracellular matrix expansion, suggesting ongoing subclinical myocardial remodeling despite the absence of overt cardiac symptoms [49]. These findings suggest that myocardial involvement in rheumatoid arthritis frequently remains clinically silent during the early stages of disease but may contribute to progressive structural and functional cardiac impairment [15,18]. Together, chronic systemic inflammation, endothelial dysfunction, coronary microvascular abnormalities, and subclinical myocardial remodeling provide a mechanistic link between rheumatoid arthritis and the increased risk of heart failure and adverse cardiovascular outcomes observed in this population [34,46,50].

These observations highlight that immune-mediated myocardial injury lies at the intersection of cardiology, rheumatology, vascular biology, and immunology and reflects a dynamic continuum linking immune activation [6,18], endothelial dysfunction, coronary microvascular impairment [15,30], oxidative stress and mitochondrial injury [16,20], and metabolic dysregulation [11,12,17] with progressive myocardial damage [19,37].

At the center of this continuum is an integrated immune–microvascular–immunometabolic axis, in which systemic inflammation induces endothelial activation and microvascular dysfunction [6,27,28,39], leading to tissue hypoxia and oxidative stress [25,30]. These processes, in turn, promote mitochondrial dysfunction [26], metabolic reprogramming [8,11,28], and activation of regulated forms of cardiomyocyte death [7,31,40,41,44,49], establishing a self-perpetuating cycle that drives progression from acute myocardial inflammation to chronic inflammatory cardiomyopathy [14,51].

The expanding availability of targeted biologic and small-molecule therapies in rheumatology creates new opportunities for mechanism-based treatment of autoimmune myocardial disease, provided that myocardial phenotyping becomes sufficiently precise [5,14,15,18,41,52]. Early recognition of myocardial inflammation is therefore critical to preventing irreversible fibrosis, adverse myocardial remodeling, and progression to inflammatory cardiomyopathy and heart failure [53,54].

This review provides an integrative overview of immune-mediated myocardial injury in systemic rheumatic diseases and proposes an immune–microvascular–immunometabolic framework as a unifying axis linking systemic autoimmunity with myocardial inflammation and progression to inflammatory cardiomyopathy [10,25,26,27,28,29,30,49]. Particular emphasis is placed on the interplay between innate and adaptive immune responses, coronary microvascular dysfunction, immunometabolic reprogramming, and emerging mechanism-based therapeutic approaches [6,7,8,26,27,28,29,39,55].

## 2. Review Methodology

This narrative review was prepared in accordance with the Scale for the Assessment of Narrative Review Articles (SANRA) framework to ensure methodological transparency and a structured synthesis of the available evidence [56].

A literature search was conducted using the PubMed/MEDLINE, Web of Science, and Scopus databases. Publications published between January 2000 and June 2026 were screened, while earlier landmark studies were retained when they provided essential historical, diagnostic, or mechanistic insights into autoimmune myocardial injury and inflammatory cardiomyopathy [24,25,43]. Particular emphasis was placed on studies published during the last decade (2015–2026), reflecting major advances in cardiovascular imaging, coronary microvascular dysfunction, immunometabolism, inflammatory cardiomyopathy, and cardio-rheumatology.

The search strategy incorporated both Medical Subject Headings (MeSH) and free-text terms related to myocardial inflammation and cardiomyopathy, including “myocarditis”, “inflammatory cardiomyopathy”, “immune-mediated myocardial injury”, “myocardial inflammation”, “heart failure”, and “cardiac involvement”, together with terms describing systemic autoimmune and rheumatic diseases, including “systemic lupus erythematosus”, “rheumatoid arthritis”, “systemic sclerosis”, “idiopathic inflammatory myopathies”, “vasculitis”, “autoimmune disease”, and “connective tissue disease”. Search terms were combined using the Boolean operators AND and OR.

Only English-language publications were considered. Eligible sources included original research articles, prospective and retrospective clinical studies, translational investigations, clinical practice guidelines, consensus statements, systematic reviews, meta-analyses, and selected narrative reviews providing relevant mechanistic insights into immune-mediated myocardial injury or documenting cardiac involvement in systemic rheumatic diseases.

Particular emphasis was placed on studies addressing coronary microvascular dysfunction innate and adaptive immune activation, immunometabolic mechanisms, cytokine signaling, oxidative stress, mitochondrial dysfunction, myocardial fibrosis, and regulated cardiomyocyte death pathways. In addition, the reference lists of key original and review articles were manually screened to identify further relevant publications.

Given the heterogeneity of the available literature and the multidisciplinary nature of the topic, this work was designed as a narrative and mechanistically oriented synthesis rather than a quantitative meta-analysis. Experimental findings derived from cellular and animal models were interpreted cautiously and integrated with clinical observations to enhance translational relevance.

Study selection was guided by relevance to the central mechanistic framework of the review, prioritizing studies that contributed to the understanding of the interplay between immune activation, vascular dysfunction, and metabolic stress in myocardial injury.

This approach was intended to provide a comprehensive, integrative, and clinically meaningful overview for both basic researchers and clinicians working in the rapidly evolving field of cardio-rheumatology.

## 3. Why the Myocardium Becomes a Target in Systemic Autoimmunity

The myocardium is particularly vulnerable in systemic autoimmune diseases because it lies at the intersection of immune surveillance, coronary microvascular dependence, high metabolic demand, and limited tolerance for sustained inflammatory remodeling [1,4,43]. Unlike tissues in which inflammation may remain spatially confined without major functional consequences, the heart is highly sensitive to disturbances in oxygen delivery, endothelial integrity, ionic homeostasis, calcium handling, and mitochondrial energy production [6,10]. Consequently, autoimmune inflammation extends beyond purely immunologic injury and encompasses hemodynamic, electrophysiologic, microvascular, and metabolic consequences that may ultimately contribute to myocardial dysfunction, fibrosis, and heart failure [1,4,43].

Several interrelated mechanisms contribute to myocardial vulnerability in systemic rheumatic diseases. A central factor is the systemic loss of immune tolerance, which permits the expansion of autoreactive T and B lymphocytes recognizing self-antigens expressed in multiple tissues, including the myocardium. In this context, the heart becomes a secondary target of systemic immune activation, even when the initiating immune response originates outside the cardiovascular system [1,42,43].

Autoimmune responses may be further amplified by molecular mimicry, in which structural similarities between exogenous antigens and host proteins initiate immune responses that subsequently cross-react with myocardial antigens. Examples include autoantibodies directed against α-myosin heavy chain, cardiac troponins, and β1-adrenergic receptors [10,43]. In parallel, epitope spreading broadens the autoimmune response over time, as ongoing tissue injury leads to the release of additional intracellular cardiac antigens, thereby sustaining chronic inflammation and facilitating the transition from acute myocarditis to inflammatory cardiomyopathy [18,43].

Post-translational modification of self-antigens represents another important mechanism. Oxidation, citrullination, carbamylation, and other inflammatory modifications generate neoepitopes that are recognized as immunogenic by the adaptive immune system [28]. Citrullination, in particular, plays a central role in rheumatoid arthritis by promoting the generation of anti-citrullinated protein antibodies (ACPAs), whereas oxidative modifications may enhance autoantigenicity and perpetuate chronic immune activation [44,50]. These processes are particularly relevant in chronic autoimmune diseases characterized by persistent inflammation and oxidative stress [45,57].

Cytokine-driven inflammation plays a central role in linking systemic immune activation with myocardial dysfunction. Proinflammatory cytokines including tumor necrosis factor-α (TNF-α), interleukin-1β (IL-1β), interleukin-6 (IL-6), and interferon-γ (IFN-γ), modulate endothelial function, leukocyte trafficking, cardiomyocyte metabolism, and extracellular matrix turnover [9,10]. Through these mechanisms, cytokine signaling may impair myocardial contractility, disrupt calcium handling, promote endothelial activation, and accelerate fibrotic remodeling [9,34].

**Figure 1:**
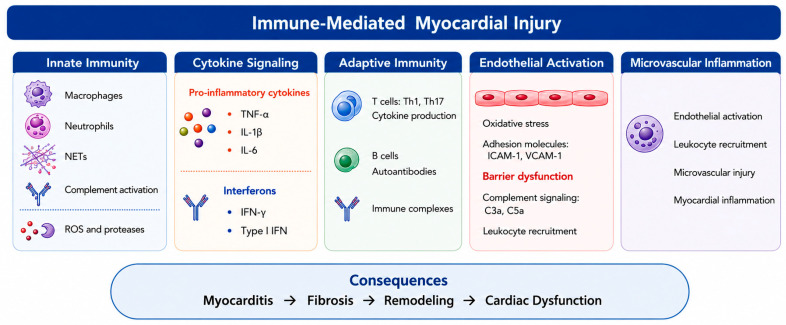
Immune–microvascular mechanisms contributing to early myocardial injury in systemic rheumatic diseases. Abbreviations: IFN, interferon; IL, interleukin; NETs, neutrophil extracellular traps; ROS, reactive oxygen species; TNF, tumor necrosis factor. Systemic autoimmune activation promotes cytokine signaling, endothelial dysfunction, coronary microvascular inflammation, leukocyte recruitment, oxidative stress, and immunometabolic reprogramming. These processes establish an immune–microvascular axis that contributes to early myocardial injury and subsequent inflammatory remodeling.

Coronary microvascular dysfunction (CMD) represents a key pathway translating systemic inflammation into myocardial injury [4,36]. Even subtle disturbances in microvascular regulation may impair myocardial perfusion, promote repeated episodes of subclinical ischemia–reperfusion injury, and increase oxidative stress, thereby contributing to progressive myocardial damage in systemic rheumatic diseases [12,39]. The resulting oxidative stress and endothelial dysfunction contribute to progressive myocardial damage in systemic rheumatic diseases [4,52]. As illustrated in Figure 1, endothelial dysfunction, coronary microvascular abnormalities, and inflammatory signaling form an interconnected immune–microvascular axis linking systemic autoimmunity with early myocardial injury.

Humoral immune mechanisms further amplify myocardial injury. Autoantibodies, immune complexes, and complement activation may directly damage endothelial cells and cardiomyocytes, while also promoting microvascular thrombosis [37]. Complement activation contributes to myocardial injury through the generation of the anaphylatoxins C3a and C5a, which recruit and activate leukocytes and enhance vascular permeability [36,37]. Formation of the membrane attack complex (C5b-9) may further induce endothelial dysfunction and microvascular damage [37].

Innate immune activation contributes to inflammatory amplification within the myocardium. Neutrophils participate in tissue injury through the release of reactive oxygen species, proteolytic enzymes, and neutrophil extracellular traps (NETs) [8,30]. NETs are extracellular DNA–protein networks decorated with histones, myeloperoxidase, and neutrophil elastase [30,33]. They promote endothelial injury, platelet activation, immunothrombosis, and microvascular dysfunction [11,30,32].

Metabolic stress constitutes an additional layer of myocardial vulnerability. In the context of systemic autoimmunity, metabolic stress refers to chronic inflammatory activation, recurrent microvascular ischemia, oxidative stress, impaired fatty acid oxidation, altered tricarboxylic acid cycle signaling, and mitochondrial dysfunction [21,22,26,27,28,29]. These processes are closely linked to immunometabolic reprogramming, whereby activated immune cells increase glycolytic flux and inflammatory metabolite production [21,22,37]. Simultaneously, cardiomyocytes experience impaired mitochondrial ATP generation and reduced energetic efficiency [26,29]. Such alterations sustain immune activation while compromising myocardial contractile performance and adaptive reserve [21,22,26,27,28,29].

Oxidative stress and mitochondrial dysfunction further amplify these processes. Excessive production of reactive oxygen species damages cellular structures and promotes inflammatory signaling, whereas mitochondrial injury may release danger-associated molecular patterns that activate innate immune pathways and perpetuate myocardial inflammation [28]. Mitochondrial injury may additionally result in the release of danger-associated molecular patterns (DAMPs), including mitochondrial DNA and oxidized mitochondrial components, which activate innate immune pathways such as NLRP3 inflammasome signaling and perpetuate myocardial inflammation [7,29]. Persistent mitochondrial dysfunction also impairs ATP generation and calcium homeostasis, thereby contributing to progressive cardiomyocyte dysfunction and adverse myocardial remodeling [26,29].

Another important feature of myocardial involvement in systemic rheumatic diseases is its frequent coexistence with multi-organ pathology. Myocardial injury often accompanies skeletal muscle inflammation, pulmonary vascular disease, interstitial lung disease, renal involvement, systemic vasculitis, and thromboinflammatory states, reflecting a shared systemic pathogenic background that influences both clinical phenotype and prognosis [1,4]. This pattern is particularly evident in systemic sclerosis, idiopathic inflammatory myopathies, systemic lupus erythematosus, and ANCA-associated vasculitis, where cardiac manifestations frequently coexist with extra-cardiac organ involvement and contribute substantially to long-term morbidity and mortality [15,18,41,42].

Clinically, immune-mediated myocardial injury may evolve along several partially overlapping trajectories. One trajectory involves acute inflammatory injury characterized by immune-cell infiltration, myocardial edema, and rapid deterioration of ventricular function [12,24]. Another reflects smoldering low-grade inflammation, in which repeated subclinical injury gradually leads to progressive fibrosis and ventricular dysfunction [34,43]. A third trajectory involves fibro-inflammatory remodeling following earlier immune-mediated or microvascular injury [24,25,43].

These trajectories are not mutually exclusive and may coexist within the same patient over time. Patients may initially develop inflammatory myocardial injury, subsequently transition to fibro-inflammatory remodeling, and ultimately present with overt inflammatory cardiomyopathy [25,26,50]. This dynamic continuum provides a conceptual framework linking early immune activation, coronary microvascular dysfunction, immunometabolic dysregulation, and chronic myocardial remodeling [4,29,36]. Understanding these interconnected mechanisms may facilitate earlier recognition of cardiac involvement in systemic autoimmune diseases and support the development of targeted therapeutic strategies aimed at preventing progression from autoimmune myocarditis to inflammatory cardiomyopathy [24,25,55].

## 4. Innate Immunity, Endothelial Activation, and Myocardial Inflammation

Innate immunity plays a central role in the initiation and amplification of myocardial inflammation in systemic autoimmune diseases. In this setting, innate immune pathways may be activated not only by infectious stimuli but also by endogenous danger-associated molecular patterns (DAMPs) released from stressed or injured cells, a process commonly referred to as sterile inflammation [7,10]. Cardiomyocytes, endothelial cells, fibroblasts, and infiltrating immune cells form a dynamic myocardial microenvironment in which immune activation, metabolic stress, and tissue injury reinforce one another, ultimately impairing myocardial function [7,10].

### 4.1. Pattern Recognition Signaling

Both immune cells and cardiac resident cells express pattern-recognition receptors, including Toll-like receptors (TLRs) and NOD-like receptors (NLRs), which detect endogenous stress signals such as extracellular nucleic acids, oxidized lipids, mitochondrial DNA, and heat-shock proteins. Activation of these receptors initiates intracellular signaling cascades that converge on nuclear factor κB (NF-κB) and interferon regulatory factors, promoting the production of proinflammatory cytokines and chemokines [11].

In systemic autoimmune diseases, innate immune signaling pathways may remain persistently activated even in the absence of infection. For example, nucleic acid–containing immune complexes in systemic lupus erythematosus activate endosomal TLR pathways and sustain type I interferon responses, thereby perpetuating immune activation and tissue injury [13,44].

The NLRP3 inflammasome has emerged as a key molecular platform linking cellular stress with myocardial inflammation. Within the myocardium, canonical NLRP3 inflammasome signaling is predominantly observed in resident and infiltrating macrophages, whereas cardiomyocytes, cardiac fibroblasts, and endothelial cells also express inflammasome-related proteins and may participate in inflammasome activation under conditions of sustained stress or injury [7,28]. Activation of inflammasome pathways results in caspase-1–dependent maturation of IL-1β and IL-18 and may trigger pyroptotic cell death, thereby amplifying myocardial inflammation and tissue injury [7,41].

Activation of innate immune signaling rapidly translates into cytokine and chemokine production within the myocardial microenvironment. Macrophages, neutrophils, endothelial cells, and activated cardiac fibroblasts release proinflammatory mediators including TNF-α, IL-1β, IL-6, IL-18, CCL2, and CXCL8, which promote leukocyte recruitment, endothelial activation, and amplification of local inflammatory responses [7,10,29]. These cytokine networks serve as a critical bridge between innate immune sensing and the development of myocardial inflammation, microvascular dysfunction, and tissue remodeling.

### 4.2. Endothelial Dysfunction as a Gatekeeper of Myocardial Injury

Coronary microvascular dysfunction (CMVD) represents one of the earliest and most important mechanisms linking systemic autoimmunity with myocardial injury. This concept is summarized in Figure 1, which illustrates how endothelial activation and microvascular dysfunction act as early mediators of myocardial injury. Endothelial activation constitutes a central component of CMVD and is characterized by upregulation of adhesion molecules such as VCAM-1 and ICAM-1, impaired vasodilatory responses, increased vascular permeability, enhanced leukocyte recruitment, and transendothelial migration. In parallel, endothelial cells may adopt a procoagulant phenotype, promoting microvascular thrombosis and impairing myocardial perfusion [35,58]. The key molecular mechanisms linking endothelial dysfunction and coronary microvascular injury with myocardial damage are summarized in Table 1.

Given the dependence of the myocardium on continuous oxygen delivery, even subtle endothelial dysfunction and CMVD may significantly reduce coronary flow reserve and predispose to patchy myocardial ischemia, repetitive ischemia–reperfusion injury, and oxidative stress. This mechanism is particularly relevant in systemic rheumatic diseases, in which microvascular dysfunction is often diffuse, persistent, and clinically silent for prolonged periods [4,35].

Advanced imaging studies have demonstrated that coronary microvascular abnormalities may occur even in the absence of obstructive epicardial coronary artery disease. In patients with autoimmune rheumatic diseases, impaired coronary microvascular function has been documented using stress cardiac magnetic resonance and positron emission tomography (PET), supporting the concept of primary microvascular involvement [51,52]. Importantly, these imaging modalities capture not only structural abnormalities but also physiological, hemodynamic, and physicochemical aspects of myocardial perfusion, including myocardial blood flow, coronary flow reserve, myocardial flow reserve, tissue oxygenation, and regional perfusion abnormalities [51,52].

Collectively, these findings indicate that CMVD and endothelial dysfunction represent central pathophysiological gateways through which systemic immune activation is translated into myocardial injury, linking vascular inflammation with microvascular ischemia, oxidative stress, metabolic dysregulation, and progressive myocardial remodeling.

### 4.3. Monocytes and Macrophages

Macrophages represent key mediators linking innate immune activation with myocardial inflammation and remodeling. Cardiac macrophages are highly heterogeneous and include both proinflammatory M1-like and reparative M2-like populations. M1-like macrophages are characterized by expression of markers such as CD80, CD86, and inducible nitric oxide synthase (iNOS) and produce proinflammatory cytokines including TNF-α, IL-1β, IL-6, and IL-12. In contrast, M2-like macrophages express markers such as CD163, CD206, and arginase-1 and contribute to resolution of inflammation, tissue repair, angiogenesis, and extracellular matrix remodeling through the release of IL-10 and transforming growth factor-β (TGF-β) [9,29].

Under physiological conditions, resident cardiac macrophages contribute to myocardial homeostasis through debris clearance, regulation of electrical conduction, maintenance of tissue integrity, and coordination of tissue repair. Recruitment of circulating monocytes to the myocardium is largely driven by chemokine pathways such as CCL2–CCR2 signaling, which are activated during tissue injury and inflammation [9,29]. Following myocardial infiltration, monocytes differentiate into macrophages and contribute to both inflammatory injury and tissue repair depending on local cytokine and metabolic cues. During autoimmune inflammation, persistent immune activation favors differentiation toward proinflammatory macrophage populations, thereby amplifying myocardial inflammation and tissue remodeling [9,29].

In autoimmune myocardial disease, sustained cytokine signaling promotes macrophage activation and polarization toward proinflammatory phenotypes. These cells release cytokines such as IL-1β, IL-6, and TNF-α,reactive oxygen species, and matrix metalloproteinases that modulate fibroblast activation and extracellular matrix turnover. Through these mechanisms, macrophages contribute not only to acute inflammatory injury but also to electrical instability, extracellular matrix remodeling, and the transition toward chronic fibro-inflammatory cardiomyopathy [29].

### 4.4. Neutrophils and NETosis

Neutrophils represent another critical component of innate immune responses in autoimmune myocardial disease [33]. Under physiological conditions, neutrophils serve as first-line effector cells of host defense through phagocytosis, degranulation, production of reactive oxygen species, and elimination of pathogens [11,33].

Neutrophils are among the earliest immune cells recruited to sites of myocardial injury and sterile inflammation. In response to chemokines, damage-associated molecular patterns (DAMPs), and endothelial activation, neutrophils rapidly infiltrate injured tissue, where they release proteolytic enzymes, reactive oxygen species, and proinflammatory mediators that amplify local immune responses. In addition, neutrophils actively interact with monocytes and macrophages, shaping subsequent inflammatory and reparative processes within the myocardium [8,11].

Beyond these classical functions, neutrophils may undergo NETosis, a specialized form of activation characterized by the release of neutrophil extracellular traps (NETs) composed of chromatin fibers, histones, and granular enzymes [32].

NETs exert multiple pathogenic effects, including direct cytotoxicity toward endothelial cells, activation of platelets, and amplification of innate immune signaling. They also provide a scaffold for thrombosis, promoting microvascular occlusion and impaired myocardial perfusion. These mechanisms are particularly relevant in systemic autoimmune diseases characterized by dysregulated NET formation [33].

NETosis has been strongly implicated in systemic lupus erythematosus, antiphospholipid syndrome, rheumatoid arthritis, and ANCA-associated vasculitis. Within the myocardium, NET formation contributes to endothelial injury, immunothrombosis, and inflammatory amplification, thereby linking vascular inflammation with myocardial damage [11,30,33].

In addition, NET-associated oxidative stress may promote oxidation of polyunsaturated fatty acids and contribute to lipid peroxidation processes involved in ferroptotic cell death. Similarly, inflammatory signaling downstream of innate immune activation may trigger pyroptosis and necroptosis, further amplifying myocardial injury, metabolic dysfunction, and persistent inflammation [28,40,41,49].

## 5. Adaptive Immunity and Autoantibody-Mediated Myocardial Injury

Although innate immune mechanisms initiate myocardial inflammation, adaptive immune responses play a central role in sustaining and shaping autoimmune myocardial injury. Activation of autoreactive T and B lymphocytes contributes to persistent inflammation, progressive myocardial remodeling, and the development of chronic inflammatory cardiomyopathy.

T lymphocytes represent a key component of autoimmune myocardial injury. Both CD4^+^ helper T cells and CD8^+^ cytotoxic T cells may infiltrate myocardial tissue and contribute to cardiomyocyte damage [3,35]. CD4^+^ T cells orchestrate inflammatory responses through cytokine production and regulation of immune-cell recruitment, whereas CD8^+^ T cells can directly induce cardiomyocyte death through perforin- and granzyme-mediated cytotoxic mechanisms [3,4,35]. In the myocardium, these processes impair contractile function, promote electrical instability, and contribute to progressive ventricular dysfunction and adverse remodeling [3,23,43].

Among CD4^+^ T-cell subsets, T helper 1 (Th1) and T helper 17 (Th17) responses are particularly relevant [10,43]. Th1-associated cytokines, including interferon-γ, promote macrophage activation and inflammatory amplification [10], whereas Th17-derived cytokines such as IL-17 enhance neutrophil recruitment and sustain chronic inflammatory tissue injury [10,43]. Together, these pathways drive persistent myocardial inflammation and facilitate the transition toward fibro-inflammatory remodeling [43]. Importantly, these cytokine-driven mechanisms are closely linked to immunometabolic reprogramming, which further sustains inflammatory activity within the myocardial microenvironment [21,22,29].

B lymphocytes also play a critical role through autoantibody production and antigen presentation. Autoantibody-mediated injury may occur through direct binding to cardiac antigens, activation of complement pathways, and formation of immune complexes that deposit within myocardial or microvascular structures [36,37,59,60,61]. In addition to structural injury, certain autoantibodies exert functional effects by interacting with cardiomyocyte or endothelial surface receptors, thereby altering intracellular signaling pathways involved in calcium handling, adrenergic signaling, and myocardial contractility [59,60,61].

Although autoantibodies are increasingly recognized as important contributors to myocardial injury, their relative pathogenic contribution likely varies across disease entities and stages of disease progression. Current evidence suggests that T-cell–mediated inflammation and innate immune activation constitute the primary drivers of myocardial injury in most forms of autoimmune myocarditis and inflammatory cardiomyopathy, whereas autoantibodies may amplify tissue damage through complement activation, immune complex formation, endothelial dysfunction, and receptor-mediated effects on cardiomyocytes. Nevertheless, in selected conditions, autoantibodies may exert direct pathogenic effects and contribute substantially to disease progression [36,37,59,60,61]. Selected downstream mechanisms linking adaptive immune activation with myocardial injury are summarized in Table 1.

In systemic lupus erythematosus, circulating immune complexes composed of autoantibodies and nuclear antigens activate complement-mediated inflammatory cascades and contribute to myocardial inflammation and dysfunction [13,44]. Type I interferon signaling represents a central pathway in SLE pathogenesis, sustaining immune activation and contributing to multi-organ involvement, including cardiovascular complications [13,44]. Imaging studies have demonstrated that myocardial involvement in SLE is frequently subclinical, with diffuse myocardial abnormalities and early fibrosis detectable using advanced techniques such as native T1 mapping [62,63].

In rheumatoid arthritis, chronic cytokine-driven inflammation and persistent immune activation promote endothelial dysfunction and myocardial remodeling. Structural and functional cardiac abnormalities, including left ventricular remodeling and subtle myocardial dysfunction, have been documented using advanced imaging techniques [45,47,48,57].

Adaptive immune mechanisms are also highly relevant in idiopathic inflammatory myopathies, where immune responses targeting striated muscle antigens may extend to the myocardium. Because cardiomyocytes share structural and antigenic features with skeletal muscle fibers, immune-mediated injury may affect both tissues simultaneously [15,16,17,18,19]. Cardiac involvement may manifest as myocarditis, arrhythmias, conduction disturbances, or progressive cardiomyopathy, often remaining clinically silent and detectable only through imaging or biomarker abnormalities [15,17,18,19,20]. Cardiac magnetic resonance imaging and circulating biomarkers, including cardiac troponins, facilitate early detection of myocardial involvement in these disorders [17,18,19,20].

Taken together, these observations indicate that adaptive immune responses not only amplify myocardial inflammation but also determine disease chronicity, myocardial remodeling, and long-term functional outcomes. The interplay between innate immune activation, adaptive immunity, and tissue-specific autoimmunity represents a central mechanism driving the transition from acute myocardial inflammation to chronic inflammatory cardiomyopathy.

## 6. Cytokine Networks and Immunometabolic Reprogramming

Cytokines represent central molecular mediators linking systemic immune activation with myocardial inflammation, cellular dysfunction, and structural remodeling in autoimmune rheumatic diseases [9,10,43]. Persistent cytokine signaling reshapes the myocardial microenvironment by modulating the behavior of cardiomyocytes, endothelial cells, cardiac fibroblasts, macrophages, and infiltrating immune cells, thereby integrating inflammatory and metabolic stress responses.

Proinflammatory cytokines such as tumor necrosis factor-α (TNF-α), interleukin-1β (IL-1β), and interleukin-6 (IL-6) play key roles in myocardial injury. These mediators promote leukocyte recruitment, increase endothelial permeability, and stimulate reactive oxygen species production. Importantly, cytokine signaling directly affects cardiomyocyte function by impairing calcium handling, reducing contractile efficiency, and disrupting excitation–contraction coupling. As a result, sustained cytokine exposure may contribute to ventricular dysfunction even in the absence of extensive structural myocardial injury [9,10,43].

Cytokine signaling also critically regulates myocardial remodeling. Transforming growth factor-β (TGF-β) is a central driver of fibroblast activation, promoting differentiation into myofibroblasts and enhancing extracellular matrix deposition. This process contributes to interstitial fibrosis, increased ventricular stiffness, and progressive impairment of both diastolic and systolic function [29,43].

Importantly, myocardial inflammation is not a static process but evolves through sequential phases of immune activation, inflammatory resolution, and tissue repair. Following the initial proinflammatory response dominated by TNF-α, IL-1β, and IL-6, anti-inflammatory pathways involving IL-10, TGF-β, and reparative macrophage populations contribute to suppression of excessive inflammation and promotion of tissue healing. The balance between persistent inflammatory activation and effective resolution is a major determinant of whether myocardial injury resolves or progresses toward chronic fibro-inflammatory remodeling and inflammatory cardiomyopathy [23,29,43].

A key feature of cytokine-driven myocardial injury is its tight integration with immunometabolic reprogramming. Activated immune cells undergo profound metabolic adaptations, including a shift toward glycolytic metabolism and altered mitochondrial activity, which sustain inflammatory effector functions. These metabolic changes are not merely adaptive but actively regulate immune-cell signaling pathways and inflammatory outputs [21,22,29].

Accumulation of metabolic intermediates further amplifies inflammation. For example, the tricarboxylic acid cycle metabolite succinate stabilizes hypoxia-inducible factor-1α (HIF-1α), thereby enhancing IL-1β production and reinforcing proinflammatory signaling. These findings illustrate how cellular metabolism directly shapes immune responses and contributes to myocardial inflammation [21]. Additional metabolites have recently emerged as important regulators of immune-cell function and inflammatory signaling. Ketone bodies, particularly β-hydroxybutyrate, may exert anti-inflammatory effects through inhibition of NLRP3 inflammasome activation and modulation of macrophage polarization. Similarly, short-chain fatty acids such as butyrate influence immune-cell differentiation, promote regulatory immune responses, and contribute to inflammatory resolution through epigenetic and metabolic mechanisms. These observations further support the concept that metabolism-derived signaling molecules actively regulate both inflammation and tissue repair [21,22,28].

Mitochondrial dysfunction represents a central node linking cytokine signaling with metabolic stress and myocardial injury. Impaired mitochondrial respiration increases reactive oxygen species production, promotes oxidative damage, and disrupts ATP generation. In cardiomyocytes, these alterations impair energy-dependent processes, including calcium homeostasis and excitation–contraction coupling, ultimately reducing contractile performance. Mitochondrial damage may also lead to the release of mitochondrial DNA and other danger-associated molecular patterns, activating innate immune pathways such as inflammasome signaling and further amplifying inflammation [17,26,28,29]. Altered NAD^+^ homeostasis may additionally compromise mitochondrial resilience and cellular stress adaptation in chronic inflammatory states [26].

Importantly, immunometabolic dysregulation extends beyond immune cells and exhibits cell-type-specific effects within the myocardium. In cardiomyocytes, inflammatory and metabolic stress impair mitochondrial energetics and excitation–contraction coupling. In endothelial cells, metabolic alterations reduce nitric oxide bioavailability, disrupt vascular tone regulation, and promote leukocyte adhesion, thereby contributing to coronary microvascular dysfunction. In cardiac fibroblasts, metabolic reprogramming promotes proliferative and profibrotic phenotypes, accelerating extracellular matrix deposition and structural remodeling. These coordinated, cell-specific responses integrate immune activation with functional and structural myocardial impairment [21,22,29]. These responses occur within a highly interconnected cellular network. Activated macrophages release cytokines that stimulate fibroblast activation and extracellular matrix deposition, while endothelial dysfunction promotes leukocyte recruitment and amplifies inflammatory signaling. In turn, stressed cardiomyocytes release danger-associated molecular patterns and metabolic signals that further activate immune cells. This bidirectional communication between immune cells, endothelial cells, fibroblasts, and cardiomyocytes creates a self-reinforcing inflammatory microenvironment that promotes persistent myocardial dysfunction and remodeling [29,43].

In addition to metabolic alterations, immunometabolic stress interacts with regulated forms of cardiomyocyte death. Oxidative stress and lipid peroxidation may trigger ferroptotic pathways, whereas inflammasome activation promotes pyroptosis, both contributing to inflammatory amplification and progressive myocardial injury [28,40,41,49]. These mechanisms represent downstream effectors linking metabolic stress with irreversible cardiomyocyte loss.

Cytokine signaling and metabolic stress also directly influence coronary microvascular function. Inflammatory mediators impair endothelial nitric oxide signaling, promote vascular inflammation, and reduce vasodilatory reserve, thereby contributing to coronary microvascular dysfunction in autoimmune diseases [4,35,39,58]. Through these pathways, immunometabolic dysregulation establishes a mechanistic bridge between systemic inflammation and impaired myocardial perfusion.

Collectively, these processes form an integrated immunometabolic network linking inflammatory signaling with mitochondrial dysfunction, endothelial impairment, and fibro-inflammatory remodeling. This network constitutes a critical component of the immune–microvascular axis driving myocardial injury and progression toward inflammatory cardiomyopathy. As illustrated in Figure 1, cytokine signaling, endothelial activation, coronary microvascular dysfunction, and metabolic stress converge to initiate early myocardial injury and sustain a self-amplifying cycle of inflammation and structural remodeling. The clinical consequences of this network depend not only on the intensity of inflammatory activation but also on the effectiveness of inflammatory resolution and tissue-repair mechanisms. The principal molecular pathways involved in this immunometabolic network are summarized in Table 1.

## 7. Oxidative Stress and Mitochondrial Injury

Oxidative stress represents a central downstream mechanism through which persistent immune activation and immunometabolic dysregulation contribute to myocardial injury in systemic rheumatic diseases. Reactive oxygen species (ROS) are generated by activated immune cells, endothelial cells, and dysfunctional mitochondria, thereby linking inflammation with metabolic and structural myocardial damage [10,26,29]. The major oxidative and mitochondrial mechanisms implicated in myocardial injury are summarized in Table 1.

**Figure 2:**
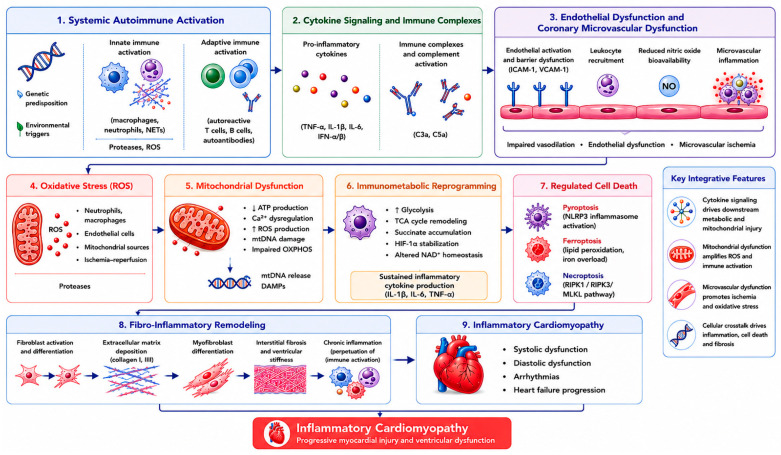
Molecular and immunometabolic cascade driving inflammatory cardiomyopathy in systemic autoimmune diseases. Abbreviations: ATP, adenosine triphosphate; DAMPs, damage-associated molecular patterns; ECM, extracellular matrix; HIF-1α, hypoxia-inducible factor-1α; IL, interleukin; mtDNA, mitochondrial DNA; NAD^+^, nicotinamide adenine dinucleotide; ROS, reactive oxygen species; TNF, tumor necrosis factor, ↑ indicates upregulation or activation; ↓ indicates downregulation or inhibition. The figure illustrates how systemic immune activation, cytokine signaling, coronary microvascular dysfunction, oxidative stress, mitochondrial injury, and immunometabolic reprogramming converge to promote regulated cardiomyocyte death, fibrosis, and progression toward inflammatory cardiomyopathy.

As illustrated in Figure 2, oxidative stress and mitochondrial dysfunction act as key integrative nodes that translate immune-mediated injury into progressive myocardial damage. Within this framework, upstream processes including cytokine signaling, coronary microvascular dysfunction, and immunometabolic reprogramming converge to disrupt mitochondrial homeostasis and cellular energetics. In this context, mitochondria function as central hubs integrating inflammatory, metabolic, and redox signaling, thereby determining the transition from reversible cellular stress to irreversible myocardial injury [26,28,29].

Within the myocardium, oxidative stress disrupts several critical biological processes. Excess ROS impairs mitochondrial respiration, alters calcium handling, and promotes lipid peroxidation of cellular membranes. These alterations reduce cardiomyocyte contractile efficiency, impair excitation–contraction coupling, and increase susceptibility to arrhythmias and cell death [20,21]. By interfering with energy-dependent ion transport and calcium cycling, oxidative stress directly compromises both systolic performance and electrical stability.

Mitochondria occupy a central role in this process. As the primary generators of ATP, they are essential for maintaining myocardial contractile function. However, mitochondrial dysfunction not only results from inflammatory injury but also amplifies it. Impaired oxidative phosphorylation leads to ATP depletion, while increased ROS production further damages mitochondrial DNA and respiratory chain components, creating a self-perpetuating cycle of oxidative injury and bioenergetic failure [26,29]. Disruption of ATP-dependent processes further aggravates calcium dysregulation and contractile dysfunction.

Mitochondrial damage may also generate endogenous danger signals that activate innate immune pathways. Mitochondrial DNA released into the cytosol or extracellular space functions as a damage-associated molecular pattern, stimulating pattern-recognition receptors and amplifying inflammatory signaling cascades [7,28]. In parallel, altered NAD^+^ homeostasis may impair mitochondrial resilience and limit the capacity of cardiomyocytes to adapt to chronic inflammatory stress [26].

In systemic rheumatic diseases, oxidative stress is driven not only by inflammatory cell activity but also by endothelial dysfunction and coronary microvascular abnormalities. Repeated episodes of subclinical ischemia and reperfusion further enhance ROS generation and exacerbate mitochondrial injury. This mechanism is particularly relevant in conditions characterized by coronary microvascular dysfunction, where impaired perfusion promotes metabolic stress and accelerates myocardial damage [4,35,39,58].

Oxidative stress also contributes to myocardial remodeling by modulating fibroblast activity and extracellular matrix turnover. ROS-dependent signaling pathways promote fibroblast proliferation, myofibroblast differentiation, and collagen deposition, thereby contributing to interstitial fibrosis, increased ventricular stiffness, and progressive structural remodeling [29,43].

In addition to structural remodeling, oxidative stress is a key trigger of regulated cardiomyocyte death. Lipid peroxidation, iron-dependent oxidative reactions, and impaired antioxidant defense systems may induce ferroptosis, while mitochondrial dysfunction and inflammatory signaling may promote additional regulated cell death pathways. These mechanisms contribute to cardiomyocyte loss, inflammatory amplification, and progressive deterioration of myocardial function [40,41,49].

Taken together, these findings indicate that oxidative stress and mitochondrial dysfunction represent central mechanisms linking immune activation, immunometabolic reprogramming, and progressive myocardial injury, ultimately driving bioenergetic failure, regulated cell death, and fibro-inflammatory remodeling in inflammatory cardiomyopathy [26,29,43].

## 8. Regulated Cell Death Pathways in Autoimmune Myocardial Injury

Beyond classical necrosis and apoptosis, regulated forms of cell death have emerged as central mechanisms driving inflammatory myocardial injury in systemic autoimmune diseases. Among these, pyroptosis, ferroptosis, and necroptosis represent key pathways linking immune activation, oxidative stress, and immunometabolic dysregulation with cardiomyocyte loss and inflammatory amplification [28,40,41,49]. As summarized in Figure 2, regulated cell death pathways arise downstream of oxidative stress, mitochondrial dysfunction, and immunometabolic dysregulation, linking immune activation with irreversible cardiomyocyte loss. The key regulated cell death pathways involved in autoimmune myocardial injury are summarized in Table 1.

Pyroptosis is a highly inflammatory form of programmed cell death mediated by inflammasome activation and caspase-1 signaling. Activation of the NOD-like receptor family pyrin domain containing 3 (NLRP3) inflammasome leads to cleavage of gasdermin proteins, formation of membrane pores, and release of proinflammatory cytokines such as IL-1β and IL-18. In the myocardium, this process not only induces cardiomyocyte injury but also amplifies local inflammatory signaling and recruits additional immune cells, thereby sustaining myocardial inflammation [7,28,41]. Pyroptosis is closely linked to upstream mechanisms discussed in previous sections, including mitochondrial dysfunction, oxidative stress, and danger-associated molecular patterns, which serve as key triggers of inflammasome activation [7,28].

Ferroptosis represents a distinct regulated cell death pathway driven by iron-dependent lipid peroxidation and impaired antioxidant defense systems. Unlike apoptosis or pyroptosis, ferroptosis is primarily characterized by oxidative membrane damage resulting from excessive lipid peroxidation. Cardiomyocytes are particularly susceptible to ferroptotic injury due to their high metabolic demand, abundant mitochondria, and reliance on tightly regulated redox balance. In conditions of chronic inflammation, mitochondrial dysfunction and reactive oxygen species accumulation promote lipid peroxidation and increase vulnerability to ferroptosis, thereby linking metabolic stress with structural myocardial damage [31,40,49].

Necroptosis represents a third regulated cell death pathway that combines features of programmed signaling with necrotic cellular injury. This pathway is mediated by receptor-interacting protein kinases (RIPK1 and RIPK3) and the pseudokinase MLKL, which disrupt cellular membranes and induce lytic, proinflammatory cell death. Necroptosis has been implicated in inflammatory cardiac disorders and may become particularly relevant when apoptotic pathways are insufficient or inhibited, thereby contributing to sustained myocardial injury and inflammatory propagation [40].

Importantly, these regulated cell death pathways do not act in isolation but form an interconnected network integrated with inflammatory signaling, oxidative stress, and immunometabolic dysregulation. As highlighted in Figure 2, mitochondrial dysfunction and redox imbalance act as central upstream drivers that converge on these cell death mechanisms, linking immune-mediated injury with irreversible cardiomyocyte loss [28,29,40,41,49].

Functionally, these pathways contribute to myocardial injury through distinct but complementary mechanisms: pyroptosis promotes inflammatory amplification, ferroptosis reflects metabolic vulnerability driven by lipid peroxidation, and necroptosis mediates lytic inflammatory injury. Together, they shape the transition from reversible cellular stress to irreversible myocardial damage and progressive structural remodeling [40,41,49].

Recognition of these pathways has important therapeutic implications. Targeting inflammasome activation, oxidative stress, lipid peroxidation, or necroptotic signaling may represent promising strategies to limit myocardial injury and interrupt the progression toward inflammatory cardiomyopathy. Importantly, the effectiveness of such interventions may depend on the dominant underlying pathogenic pathway, highlighting the need for mechanism-based therapeutic approaches in autoimmune myocardial disease [7,28,40,49,55].

## 9. Coronary Microvascular Dysfunction in Autoimmune Myocardial Disease

Coronary microvascular dysfunction (CMD) has emerged as a central mechanism linking systemic autoimmune inflammation with myocardial injury. Unlike obstructive epicardial coronary artery disease, CMD involves both structural and functional abnormalities of the coronary microcirculation, including endothelial dysfunction, impaired vasodilatory reserve, microvascular rarefaction, and microvascular thrombosis [4,35,39,58].

Given the high metabolic demand of the myocardium and its dependence on continuous oxygen delivery, even subtle disturbances in microvascular integrity may significantly impair cardiomyocyte perfusion [4,35,39]. Chronic impairment of microvascular flow may therefore lead to repeated episodes of subclinical ischemia–reperfusion injury, promoting oxidative stress, mitochondrial dysfunction, and inflammatory amplification, as discussed in previous sections [4,29,39].

Endothelial activation represents a central driver of CMD in systemic autoimmune diseases. Inflammatory cytokines, immune complexes, oxidative stress, and complement activation induce endothelial dysfunction characterized by reduced nitric oxide bioavailability, increased expression of adhesion molecules, and enhanced leukocyte recruitment [35]. These changes disrupt vascular homeostasis and impair coronary flow regulation [4,35,39].

Humoral immune mechanisms further contribute to microvascular injury. In systemic lupus erythematosus, antiphospholipid antibodies and immune complexes promote endothelial activation and microvascular thrombosis, thereby impairing myocardial perfusion despite angiographically normal epicardial coronary arteries [13,14,38,44]. Clinical imaging studies have confirmed the presence of myocardial ischemia in such patients, supporting the central role of microvascular dysfunction in myocardial injury [14,46,52].

In systemic sclerosis, CMD represents a hallmark of disease pathophysiology. Progressive endothelial injury leads to capillary rarefaction, structural remodeling of small vessels, and impaired vasodilatory responses. These alterations result in chronic myocardial ischemia and contribute to diffuse interstitial fibrosis and ventricular dysfunction [16,42].

Importantly, CMD is now recognized as a shared cardiovascular phenotype across multiple autoimmune rheumatic diseases [4,35,58]. Chronic inflammation, endothelial dysfunction, and vascular remodeling collectively impair coronary microvascular function and contribute to myocardial injury, irrespective of the underlying disease entity [4,35,58].

Advances in multimodal cardiac imaging have significantly improved the detection and characterization of CMD. Techniques such as stress cardiac magnetic resonance imaging, positron emission tomography, and invasive coronary flow reserve assessment allow detailed evaluation of microvascular function and myocardial perfusion abnormalities, facilitating earlier diagnosis of subclinical myocardial involvement [3,4,14,52].

Beyond being a consequence of systemic inflammation, CMD may actively drive myocardial disease progression. Repeated microvascular ischemia promotes cardiomyocyte stress responses, stimulates fibroblast activation, and amplifies inflammatory signaling, thereby linking vascular dysfunction with myocardial remodeling and progression toward inflammatory cardiomyopathy [24,29,43].

As summarized in Table 1, coronary microvascular dysfunction arises from the interplay of endothelial dysfunction, oxidative stress, immunometabolic reprogramming, mitochondrial injury, and regulated cell death pathways [4,21,29,35,39,58]. These mechanisms converge to impair coronary flow reserve, disrupt myocardial perfusion, and establish a self-perpetuating immune–microvascular–metabolic cycle driving progressive myocardial injury and inflammatory cardiomyopathy.

## 10. Disease-Specific Patterns of Myocardial Involvement in Rheumatic Diseases

Although several pathogenic pathways are shared across systemic autoimmune disorders, their relative contribution varies substantially between diseases, resulting in distinct myocardial injury phenotypes. To facilitate cross-disease comparison, the dominant immune mechanisms, molecular drivers, microvascular and myocardial injury pathways, and characteristic cardiac manifestations are summarized in Table 2.

### 10.1. Systemic Lupus Erythematosus

Systemic lupus erythematosus (SLE) represents one of the most extensively studied autoimmune diseases in the context of cardiovascular involvement. While pericarditis and accelerated atherosclerosis are well-recognized, myocardial injury is increasingly acknowledged as a clinically relevant and often underdiagnosed manifestation [13,44,63].

Multiple mechanisms contribute to myocardial involvement in SLE. Immune complex deposition and complement activation play central roles in initiating inflammatory injury within vascular and myocardial tissues. Circulating immune complexes composed of autoantibodies and nuclear antigens activate complement cascades, promoting endothelial injury and myocardial inflammation [13,37,44].

Type I interferon signaling represents a key pathogenic axis in SLE and contributes to sustained immune activation, endothelial dysfunction, and inflammatory amplification within the myocardium [13,44]. In addition, antiphospholipid antibodies may promote microvascular thrombosis and impair myocardial perfusion, linking humoral immunity with coronary microvascular dysfunction [14,38,44].

Cardiac magnetic resonance (CMR) studies have demonstrated that myocardial involvement in SLE is frequently subclinical and may include myocardial edema, diffuse fibrosis, and impaired myocardial strain, even in patients without overt cardiovascular symptoms [62,63]. These findings highlight the importance of advanced imaging for early detection of myocardial injury.

### 10.2. Rheumatoid Arthritis

Rheumatoid arthritis (RA) is primarily characterized by chronic synovial inflammation; however, systemic immune activation extends beyond the joints and significantly affects the cardiovascular system. While accelerated atherosclerosis has traditionally been emphasized, increasing evidence supports the presence of direct myocardial involvement [34,50].

Persistent cytokine-driven inflammation, particularly mediated by TNF-α and IL-6, represents a major driver of cardiovascular pathology in RA. These cytokines promote endothelial dysfunction, oxidative stress, and alterations in myocardial metabolism, thereby contributing to ventricular remodeling and impaired myocardial function [34,50,57].

Advanced imaging studies have demonstrated structural and functional myocardial abnormalities in RA patients, including left ventricular remodeling and subclinical myocardial dysfunction [45,47,48,57]. These findings support the concept that chronic systemic inflammation contributes to myocardial injury even in the absence of overt cardiac disease.

Recognition of cardiovascular involvement in RA has led to international recommendations emphasizing systematic cardiovascular risk assessment and aggressive control of systemic inflammation [1,50].

### 10.3. Systemic Sclerosis

Systemic sclerosis (SSc) is characterized by a combination of immune dysregulation, microvascular disease, and progressive fibrosis. Among systemic rheumatic diseases, SSc is particularly notable for its prominent microvascular pathology, which directly impacts myocardial perfusion [16,42].

Endothelial injury represents an early and central pathogenic event. Progressive microvascular damage leads to capillary rarefaction, impaired coronary flow reserve, and reduced tissue perfusion. Recurrent episodes of ischemia–reperfusion injury generate oxidative stress and promote fibroblast activation, ultimately leading to diffuse myocardial fibrosis [16,29,42].

Cardiac involvement in SSc may manifest as silent myocardial fibrosis, ventricular dysfunction, arrhythmias, or conduction abnormalities. Importantly, myocardial injury is frequently subclinical in early stages, requiring advanced imaging techniques such as CMR or PET for detection [3,16,42]. These findings underscore the importance of early cardiovascular screening and monitoring in patients with SSc.

### 10.4. Idiopathic Inflammatory Myopathies

Idiopathic inflammatory myopathies (IIM), including polymyositis and dermatomyositis, are characterized by immune-mediated skeletal muscle injury that may extend to the myocardium due to shared structural and antigenic features of striated muscle [15,18,19].

Cardiac involvement in IIM may manifest as myocarditis, arrhythmias, conduction disturbances, or progressive cardiomyopathy. Cytotoxic T-cell–mediated injury and autoantibody-driven mechanisms contribute to cardiomyocyte damage and inflammatory remodeling [15,18,19].

Cardiovascular magnetic resonance imaging has emerged as a key tool for detecting myocardial inflammation and fibrosis, even in asymptomatic patients [17,18,20]. Additional studies have identified clinical predictors of cardiac involvement, emphasizing the need for systematic cardiovascular assessment in this population [20].

Serological biomarkers, particularly cardiac troponins, may provide supportive evidence of myocardial injury and disease activity [17,19,20].

### 10.5. Vasculitis and Eosinophilic Myocardial Disease

Systemic vasculitides represent a heterogeneous group of disorders in which inflammation of small and medium-sized vessels may compromise myocardial perfusion and lead to ischemic or inflammatory myocardial injury [1,58].

Among these conditions, eosinophilic granulomatosis with polyangiitis (EGPA) is particularly notable for its strong association with cardiac involvement. Eosinophil-mediated cytotoxicity, driven by the release of granule proteins, may cause myocardial necrosis, inflammation, and fibrotic remodeling [65,66].

Necrotizing vasculitis, ANCA-associated immune activation, and eosinophilic infiltration collectively contribute to myocardial injury through both vascular and direct cardiotoxic mechanisms [1,65,66].

Cardiac involvement in EGPA represents a major determinant of prognosis and mortality. Early recognition of myocardial injury is therefore critical, as prompt initiation of immunosuppressive therapy may prevent irreversible cardiac damage [65,66].

## 11. Diagnostic Strategies in Autoimmune Myocardial Disease

Early recognition of myocardial involvement in systemic rheumatic diseases remains a major clinical challenge. Clinical manifestations are frequently nonspecific and may include fatigue, exertional dyspnea, chest discomfort, or palpitations. Importantly, myocardial inflammation may remain clinically silent for prolonged periods and often becomes apparent only after the development of structural remodeling or ventricular dysfunction, contributing to delayed diagnosis and adverse outcomes [1,3].

Traditional diagnostic tools such as electrocardiography and transthoracic echocardiography represent essential first-line assessments but have limited sensitivity for detecting early or diffuse myocardial inflammation. Electrocardiographic abnormalities are often nonspecific and may include ST-segment changes, conduction disturbances, or arrhythmias. Echocardiography may identify ventricular dysfunction, diastolic abnormalities, or regional wall-motion abnormalities, but typically reflects more advanced stages of myocardial involvement [1,3].

Cardiac magnetic resonance (CMR) imaging has emerged as a cornerstone noninvasive modality for the evaluation of inflammatory myocardial disease. Contemporary CMR techniques enable comprehensive tissue characterization, including detection of myocardial edema, hyperemia, necrosis, and fibrosis. The updated Lake Louise criteria integrate T1- and T2-based imaging parameters, significantly improving diagnostic accuracy for myocarditis and inflammatory cardiomyopathy [2].

CMR is particularly valuable in autoimmune myocardial disease due to its ability to detect diffuse and subclinical myocardial abnormalities. Quantitative mapping techniques, including native T1, T2, and extracellular volume (ECV), provide sensitive markers of myocardial inflammation and interstitial fibrosis, even in the absence of overt ventricular dysfunction [3,17,62,63]. These techniques are especially relevant in systemic rheumatic diseases, where myocardial involvement is often patchy, subtle, and temporally heterogeneous.

Additional imaging modalities may provide complementary insights into disease activity. Positron emission tomography (PET) imaging allows detection of metabolically active inflammatory processes through increased glucose uptake in activated immune cells. This approach may help distinguish active inflammation from chronic fibrotic remodeling and may be particularly useful in selected inflammatory cardiomyopathies or systemic inflammatory conditions [3,16,42,52].

Biomarkers represent another important component of diagnostic evaluation. Cardiac troponins, particularly high-sensitivity assays, are sensitive indicators of myocardial injury and may reflect ongoing inflammatory damage, although they lack specificity for immune-mediated mechanisms. Natriuretic peptides, including B-type natriuretic peptide (BNP) and N-terminal pro-BNP (NT-proBNP), provide information on myocardial stress and ventricular dysfunction and may carry prognostic significance. Inflammatory markers such as C-reactive protein (CRP) and erythrocyte sedimentation rate (ESR) may support the presence of systemic inflammation but are not specific for myocardial involvement [1,53,54].

Endomyocardial biopsy remains the reference standard for definitive diagnosis of myocarditis and inflammatory cardiomyopathy. Histopathological and immunohistochemical analyses enable characterization of inflammatory infiltrates, identification of immune-cell subtypes, and detection of cardiomyocyte injury. However, due to the focal and heterogeneous nature of myocardial inflammation, biopsy sensitivity may be limited. Moreover, the invasive nature of the procedure restricts its use to selected clinical scenarios in which diagnostic clarification is expected to influence management decisions [1].

Given these limitations, contemporary diagnostic strategies increasingly rely on a multimodal and integrative approach that combines clinical assessment, advanced imaging techniques, biomarker evaluation, and disease-specific rheumatologic context [1,3,53]. Importantly, the identification of myocardial involvement should be interpreted within a broader pathophysiological framework that considers underlying immune mechanisms, microvascular dysfunction, and immunometabolic alterations, as discussed in previous sections. Such a phenotype–endotype-oriented approach may facilitate earlier detection of subclinical disease, improve risk stratification, and support the development of mechanism-based therapeutic strategies in autoimmune myocardial disease.

## 12. Biomarkers and Risk Stratification

Biomarkers play an increasingly important role in the detection, monitoring, and risk stratification of autoimmune myocardial disease. Although no single biomarker is specific for inflammatory cardiomyopathy, circulating markers may provide valuable information regarding cardiomyocyte injury, inflammatory activity, myocardial stress, and fibrotic remodeling. When interpreted in conjunction with imaging findings and clinical context, biomarkers may facilitate earlier detection of subclinical myocardial involvement and improve prognostic assessment [1,53].

Cardiac troponins, particularly high-sensitivity assays (hs-cTn), represent the most widely used biomarkers of myocardial injury. Elevated troponin concentrations reflect cardiomyocyte damage and may be observed in both acute myocarditis and chronic inflammatory cardiomyopathy. In patients with systemic autoimmune diseases, even mild or persistent elevations may indicate ongoing subclinical myocardial injury and should prompt further evaluation using advanced cardiac imaging modalities such as cardiac magnetic resonance [1,17,19,53].

Natriuretic peptides, including B-type natriuretic peptide (BNP) and N-terminal pro-BNP (NT-proBNP), reflect myocardial wall stress and neurohormonal activation. These biomarkers are widely used for the assessment of ventricular dysfunction and heart failure severity. In autoimmune myocardial disease, elevated natriuretic peptide levels are associated with adverse outcomes and may provide important prognostic information, even in the absence of overt clinical heart failure [53,67,68].

Several emerging biomarkers may further enhance risk stratification in inflammatory cardiac disorders. Growth differentiation factor-15 (GDF-15), soluble suppression of tumorigenicity-2 (sST2), and galectin-3 are associated with myocardial fibrosis, inflammation, and extracellular matrix remodeling. Elevated levels of these biomarkers have been linked to worse cardiovascular outcomes and may provide additional insights into disease progression in inflammatory cardiomyopathy [53,54].

Inflammatory biomarkers such as C-reactive protein (CRP) and erythrocyte sedimentation rate (ESR) reflect systemic immune activation but lack specificity for myocardial involvement. Nevertheless, persistently elevated inflammatory markers may indicate active autoimmune disease and indirectly suggest an increased risk of myocardial injury, particularly when accompanied by cardiac symptoms or abnormalities on imaging studies [1,44].

Autoantibody profiles may provide additional mechanistic insights and support risk stratification. Antiphospholipid antibodies are associated with increased risk of microvascular thrombosis and endothelial dysfunction, whereas selected myositis-specific antibodies have been linked to a higher prevalence of cardiac involvement in idiopathic inflammatory myopathies. These immunological markers may therefore indicate a higher likelihood of myocardial injury and help define disease-specific risk profiles [14,15,19,38].

Emerging evidence also highlights the potential role of novel immunometabolic and molecular biomarkers, including circulating microRNAs, markers of oxidative stress, and indicators of mitochondrial dysfunction. Although these biomarkers remain largely investigational, they may contribute to more precise phenotyping of autoimmune myocardial disease and identification of specific pathophysiological endotypes in the future [21,26,29].

Importantly, no single biomarker reliably distinguishes inflammatory myocardial injury from other causes of cardiac dysfunction. Therefore, biomarker interpretation must be integrated with imaging findings and the broader clinical and rheumatologic context. A multimodal strategy combining biomarkers, advanced cardiac imaging, and disease-specific assessment currently represents the most effective approach for risk stratification [1,3,53].

From a clinical perspective, biomarker-based risk stratification may be conceptualized along a continuum. Patients with isolated mild biomarker abnormalities may represent an early or subclinical stage of myocardial involvement, whereas those with combined elevation of cardiac injury markers, natriuretic peptides, and inflammatory biomarkers are more likely to exhibit active myocardial inflammation and an increased risk of progression toward inflammatory cardiomyopathy.

## 13. Therapeutic Perspectives

Management of autoimmune myocardial disease remains challenging, as effective treatment must simultaneously address systemic immune dysregulation and myocardial injury. Accordingly, therapeutic strategies typically combine immunomodulatory approaches targeting the underlying rheumatic disease with guideline-directed cardiovascular therapies aimed at preserving myocardial structure and function [1,55].

### 13.1. Glucocorticoids

Corticosteroids remain a cornerstone of therapy for many forms of inflammatory myocardial involvement, particularly in acute autoimmune myocarditis and inflammatory cardiomyopathy. Their broad anti-inflammatory effects include suppression of proinflammatory cytokine signaling, inhibition of leukocyte recruitment, and attenuation of immune-mediated tissue injury. However, long-term corticosteroid use is associated with substantial adverse effects, necessitating the use of steroid-sparing strategies whenever possible [1,15,55]. Beyond their broad anti-inflammatory effects, glucocorticoids may rapidly reduce myocardial edema, suppress inflammatory cell infiltration, and improve ventricular function in acute immune-mediated myocardial injury [1,55].

### 13.2. Conventional Immunosuppressive Therapy

Additional immunosuppressive agents, including azathioprine, methotrexate, mycophenolate mofetil, and cyclophosphamide, are widely used in systemic autoimmune diseases. These therapies target key components of adaptive immune responses and may help control both systemic inflammation and myocardial involvement, particularly in diseases such as systemic lupus erythematosus and idiopathic inflammatory myopathies [13,15,16,55].

Methotrexate, azathioprine, mycophenolate mofetil, and cyclophosphamide are frequently used as steroid-sparing therapies. Beyond controlling systemic inflammation, these agents may reduce endothelial activation, attenuate immune-mediated myocardial injury, and limit progression toward chronic fibro-inflammatory remodeling [13,15,16,55].

### 13.3. Biologic Therapies

The development of targeted biologic therapies has significantly expanded treatment options in rheumatology and offers promising implications for autoimmune myocardial disease. Agents targeting tumor necrosis factor-α, interleukin-6 signaling, and B lymphocytes have transformed the management of several systemic inflammatory conditions. By reducing systemic inflammatory burden and modulating key cytokine pathways, these therapies may indirectly improve myocardial inflammation and coronary microvascular dysfunction [6,34,50,55]. Biologic agents may exert cardiovascular benefits beyond suppression of systemic inflammation. TNF-α and IL-6 inhibition may improve endothelial function, reduce oxidative stress, and mitigate coronary microvascular dysfunction, whereas B-cell depletion may reduce autoantibody-mediated myocardial injury [6,34,50,55].

### 13.4. Targeted Therapies

Emerging therapies targeting specific immune pathways, including Janus kinase (JAK) inhibitors and other pathway-specific agents, may further refine treatment by modulating intracellular signaling networks involved in immune activation and inflammation. However, their role in autoimmune myocardial involvement remains incompletely defined and requires further investigation. JAK inhibitors and other pathway-specific therapies may influence intracellular inflammatory signaling, immunometabolic pathways, and cytokine networks involved in myocardial injury [21,22,26].

### 13.5. Cardiovascular and Heart Failure Therapy

Contemporary guideline-directed medical therapy remains essential in patients with inflammatory cardiomyopathy and ventricular dysfunction. ACE inhibitors and ARNI improve ventricular remodeling and neurohormonal balance, whereas β-blockers reduce arrhythmic risk and myocardial oxygen demand. Mineralocorticoid receptor antagonists attenuate myocardial fibrosis and adverse remodeling. Sodium-glucose cotransporter-2 inhibitors have emerged as particularly attractive agents because, in addition to reducing heart failure hospitalizations and mortality, they may exert favorable metabolic, anti-inflammatory, and endothelial effects [55,67,68].

In addition to pharmacological therapy, management of arrhythmias and prevention of sudden cardiac death are critical components of care. Device-based therapies, including implantable cardioverter-defibrillators and cardiac resynchronization therapy, may be indicated in selected patients with advanced disease and electrical instability [55,67,68].

### 13.6. Emerging Mechanism-Based Therapies

Future therapeutic strategies increasingly focus on targeted modulation of key pathogenic pathways identified in autoimmune myocardial disease. These include inhibition of inflammasome signaling, modulation of immunometabolic pathways, reduction in oxidative stress, and targeting of regulated cell death mechanisms such as ferroptosis and pyroptosis [7,21,26,28,31,40,41,49].

Given the central role of coronary microvascular dysfunction in linking systemic inflammation with myocardial injury, therapies aimed at improving endothelial function and restoring microvascular perfusion may also represent important adjunctive strategies. Interventions targeting nitric oxide signaling, oxidative stress pathways, and mitochondrial function may help interrupt the cycle of microvascular and myocardial injury described in previous sections [4,26,29,39].

### 13.7. Integrated and Personalized Therapeutic Approach

Given the heterogeneity of autoimmune myocardial disease, optimal management requires an individualized and mechanism-based approach. Integration of clinical presentation, imaging findings, biomarker profiles, and underlying rheumatologic disease allows identification of dominant pathophysiological processes, including inflammatory, microvascular, immunometabolic, or fibroproliferative endotypes.

Early recognition and close longitudinal monitoring are essential, as myocardial involvement may progress despite minimal symptoms. Regular assessment using advanced imaging and biomarkers enables timely therapeutic adjustment and may improve long-term outcomes.

Future therapeutic strategies may increasingly rely on mechanism-based phenotyping, enabling selection of targeted therapies tailored to dominant inflammatory, microvascular, or immunometabolic endotypes, thereby supporting the transition toward precision medicine in autoimmune myocardial disease [1,55].

## 14. Future Directions in Cardio-Rheumatology

Despite substantial advances in understanding immune-mediated myocardial injury, many aspects of autoimmune myocardial disease remain insufficiently defined. Several key areas require further investigation in order to improve diagnosis, risk stratification, and therapeutic strategies in patients with systemic rheumatic diseases.

One of the major challenges is the early identification of myocardial involvement. Because clinical manifestations are often subtle or nonspecific, myocardial inflammation may remain undetected until structural remodeling and ventricular dysfunction have already developed. Improved screening strategies integrating multimodal imaging, circulating biomarkers, and disease-specific risk profiling may therefore play a critical role in enabling earlier detection of cardiac involvement and preventing irreversible myocardial damage [1,3,53].

Another important research direction involves more precise characterization of disease endotypes. Autoimmune myocardial disease likely represents a heterogeneous spectrum of conditions with distinct biological drivers, including immune complex–mediated inflammation, coronary microvascular dysfunction, cytokine-driven injury, and fibro-inflammatory remodeling. Identification of dominant pathogenic pathways in individual patients may enable mechanism-based therapeutic selection and improve clinical outcomes [4,29,43].

Advances in molecular and cellular profiling technologies offer further opportunities to refine disease characterization. High-throughput approaches such as transcriptomics, proteomics, and metabolomics may provide deeper insight into the molecular networks underlying autoimmune myocardial injury. These techniques may facilitate identification of novel biomarkers, improve disease classification, and support development of targeted therapeutic strategies.

Immunometabolism represents another emerging field of particular relevance. Increasing evidence indicates that metabolic reprogramming of immune cells and cardiac resident cells contributes to persistent inflammation, mitochondrial dysfunction, and myocardial remodeling. Targeting key metabolic pathways involved in immune activation may therefore represent a promising therapeutic strategy in inflammatory cardiomyopathy [21,22,26,29].

Integration of cardiology and rheumatology expertise will be essential for advancing both research and clinical care. Multidisciplinary collaboration between cardiologists, rheumatologists, immunologists, and imaging specialists may improve early recognition of myocardial involvement, enable more precise phenotyping, and facilitate coordinated, mechanism-based treatment strategies.

Finally, prospective clinical studies are critically needed to establish evidence-based management of autoimmune myocardial disease. Most current therapeutic approaches are derived from small observational studies or extrapolated from systemic rheumatic disease management. Large prospective cohorts and randomized clinical trials will be required to validate diagnostic algorithms, refine risk stratification, and define optimal therapeutic strategies in patients with inflammatory cardiomyopathy.

Future progress in this field will likely depend on the integration of multimodal diagnostics, molecular profiling, and mechanism-based therapeutic approaches, ultimately enabling a transition toward precision medicine in autoimmune myocardial disease [1,55].

## 15. Conclusions

Immune-mediated myocardial injury represents an important yet still underrecognized manifestation of systemic rheumatic diseases. Rather than constituting a single pathological entity, autoimmune myocardial disease encompasses a heterogeneous spectrum ranging from acute myocarditis to chronic low-grade inflammation, fibro-inflammatory remodeling, and the development of inflammatory cardiomyopathy [3,4].

Across this spectrum, several interconnected biological processes consistently emerge, including endothelial dysfunction and coronary microvascular injury, activation of innate and adaptive immune responses, and immunometabolic dysregulation. These pathways interact within the myocardial microenvironment to create a self-reinforcing network linking systemic immune activation with local tissue injury and progressive structural remodeling [4,21,29,43].

A central concept arising from this review is the existence of an integrated immune–microvascular–immunometabolic continuum, in which systemic inflammation induces endothelial dysfunction and coronary microvascular impairment, leading to tissue hypoxia, oxidative stress, and mitochondrial injury. These processes drive metabolic reprogramming and activate regulated forms of cardiomyocyte death, including pyroptosis, ferroptosis, and necroptosis, ultimately resulting in irreversible cardiomyocyte loss and progressive ventricular dysfunction [21,26,28,31,40,41,49].

Importantly, myocardial involvement reflects not only inflammatory cell infiltration but also the convergence of immune, vascular, and metabolic disturbances that together determine disease progression and clinical phenotype. This integrated framework provides a mechanistic basis for understanding how early, potentially reversible myocardial injury evolves into chronic inflammatory cardiomyopathy characterized by fibrosis, electrical instability, and heart failure [29,43].

Recent advances in multimodal cardiac imaging, biomarker profiling, and targeted immunomodulatory therapies have substantially improved the detection and characterization of autoimmune myocardial disease. Nevertheless, early-stage myocardial involvement remains frequently underdiagnosed, highlighting the need for improved screening strategies and greater clinical awareness [1,3,62,63].

From a translational perspective, the identification of dominant pathogenic pathways—including inflammatory, microvascular, and immunometabolic endotypes—may enable more precise risk stratification and support the development of mechanism-based therapeutic strategies. Such an approach is essential for moving beyond generalized immunosuppression toward personalized treatment in cardio-rheumatology [1,55].

In conclusion, immune-mediated myocardial injury should be conceptualized as a dynamic, multi-level process integrating immune activation, coronary microvascular dysfunction, immunometabolic stress, and regulated cardiomyocyte death. Bridging mechanistic insights with clinical phenotyping will be critical for enabling earlier diagnosis, improving risk stratification, and advancing precision medicine approaches in inflammatory cardiomyopathy associated with systemic autoimmune diseases [1,43,55].

## Figures and Tables

**Table 1:** Mechanistic pathways linking systemic autoimmunity, coronary microvascular dysfunction, and inflammatory cardiomyopathy.

Dominant Pathophysiological Axis	Key Molecular Drivers	Cellular and Molecular Consequences	Microvascular Structural/Functional Consequence	Impact on Myocardial Perfusion and CFR	Clinical Correlates/Imaging Signature	Candidate Therapeutic Targets
NO signaling dysregulation [4,35,39]	eNOS uncoupling, BH4 depletion, ↑ ADMA, ↓ KLF2/4	Reduced NO bioavailability, ↑ superoxide, endothelial activation	Impaired vasodilation, ↑ leukocyte adhesion	↓ CFR, hypoperfusion	Reduced CFR on PET/CMR	BH4 restoration, AMPK activation
Oxidative stress axis [10,29,42]	NADPH oxidase, mitochondrial ROS, Nrf2 dysregulation	Lipid peroxidation, redox imbalance	Endothelial dysfunction	Impaired dilation	CMR T1/T2 changes	Nrf2 activation
Immunometabolic reprogramming [21,22,29]	HIF-1α, succinate, PKM2, mTOR	Glycolytic shift, IL-1β inflammation	Chronic endothelial inflammation	↓ CFR	FDG-PET uptake	AMPK, mTOR inhibitors
Mitochondrial dysfunction [26,28,29]	mtROS, impaired mitophagy	Bioenergetic failure, DAMPs	Reduced endothelial resilience	Impaired energetics	Strain imaging changes	Mitochondrial therapies
ER stress activation [41]	PERK, IRE1α, ATF6	Endothelial stress	Capillary rarefaction	Perfusion impairment	CMR fibrosis	ER stress modulation
Pyroptosis [7,28,41]]	NLRP3, caspase-1	Inflammatory cell death	Barrier disruption	Microvascular dysfunction	T2 edema	IL-1 blockade
Ferroptosis [31,49]	Iron overload, GPX4 dysfunction	Oxidative damage	Rarefaction	Reduced perfusion	Troponin ↑	Iron modulation
Necroptosis [40]	RIPK1/RIPK3	Inflammatory necrosis	Vascular inflammation	Perfusion instability	Cardiomyopathy	RIPK inhibitors
Immunothrombosis [11,30,32,38]	NETs, complement	Microthrombi	Capillary obstruction	Ischemia	Perfusion defects	Antithrombotic therapies

Abbreviations: ADMA—asymmetric dimethylarginine; AMPK—AMP-activated protein kinase; ATF6—activating transcription factor 6; BH4—tetrahydrobiopterin; CFR—coronary flow reserve; CMR—cardiac magnetic resonance; DAMPs—damage-associated molecular patterns; eNOS—endothelial nitric oxide synthase; ER—endoplasmic reticulum; FDG—fluorodeoxyglucose; GPX4—glutathione peroxidase 4; HIF-1α—hypoxia-inducible factor-1α; IL—interleukin; IRE1α—inositol-requiring enzyme 1α; KLF—Krüppel-like factor; mTOR—mechanistic target of rapamycin; mtROS—mitochondrial reactive oxygen species; NETs—neutrophil extracellular traps; NO—nitric oxide; Nrf2—nuclear factor erythroid 2-related factor 2; PERK—protein kinase RNA-like ER kinase; PET—positron emission tomography; PKM2—pyruvate kinase M2; RIPK—receptor-interacting protein kinase; ROS—reactive oxygen species, ↑ indicates elevated levels; ↓ indicates reduced levels.

**Table 2:** Immunopathogenic mechanisms and cardiac manifestations of immune-mediated myocardial injury across systemic rheumatic diseases.

Rheumatic Disease	Dominant Immune Mechanisms	Key Molecular Drivers	Microvascular/Myocardial Injury Pathways	Typical Cardiac Phenotype	Diagnostic Clues and Therapeutic Considerations
Systemic lupus erythematosus (SLE)	Immune complex deposition, complement activation, and persistent type I interferon signaling drive vascular and myocardial inflammation [13,37,44].	Complement activation, antiphospholipid antibodies, IFN-I axis, IL-6 and TNF-α signaling promote endothelial activation and immunometabolic stress [13,38,44].	Endothelial dysfunction and coronary microvascular dysfunction result in recurrent subclinical ischemia and inflammatory myocardial injury [14,35,52].	Subclinical myocarditis, diffuse myocardial fibrosis, arrhythmias, ventricular dysfunction [62,63].	CMR with T1/T2 mapping detects edema and diffuse fibrosis; mild troponin elevation reflects myocardial injury. Targeted control of systemic inflammation may limit cardiac involvement [44,63].
Rheumatoid arthritis (RA)	Chronic cytokine-driven immune activation dominated by TNF-α and IL-6 sustains systemic inflammation and vascular injury [34,50].	NF-κB activation, oxidative stress, cytokine-mediated signaling, and metabolic remodeling promote endothelial dysfunction [34,50,64].	Inflammation-induced endothelial dysfunction and CMD promote myocardial remodeling and metabolic stress [35,46].	Left ventricular remodeling, subclinical myocardial dysfunction, increased heart failure risk [45,47,48,57].	CMR detects fibrosis and remodeling; aggressive anti-inflammatory and targeted therapies may improve cardiovascular outcomes [45,47,48,57,64].
Systemic sclerosis (SSc)	Immune dysregulation and endothelial injury drive fibro-inflammatory microvasculopathy and progressive fibrosis [16,42].	TGF-β signaling, endothelin-1 pathways, oxidative stress, and ischemia–reperfusion-related mitochondrial injury [16,29,42].	Severe CMD and repeated ischemia–reperfusion injury result in diffuse myocardial fibrosis and microvascular rarefaction [4,16,42].	Silent myocardial fibrosis, ventricular dysfunction, conduction abnormalities [3,16,42].	Stress CMR or PET detect perfusion abnormalities and fibrosis; early cardiovascular screening is critical for disease management [3,16,42].
Idiopathic inflammatory myopathies (IIM)	Autoimmune responses against striated muscle antigens involving T- and B-cell-mediated mechanisms [15,18,19].	Autoantibody production, cytotoxic T-cell activity, and inflammatory signaling pathways drive myocardial inflammation [15,18,19].	Direct cardiomyocyte injury and inflammatory infiltration result in myocarditis and fibrotic remodeling [17,18,20].	Myocarditis, arrhythmias, conduction disturbances, inflammatory cardiomyopathy [17,18,19,20].	Troponin elevation indicates myocardial injury; CMR detects inflammation and fibrosis. Early immunosuppressive therapy improves outcomes [17,18,19,20].
Systemic vasculitides and EGPA	Necrotizing vascular inflammation with neutrophil activation and eosinophil-mediated toxicity drives myocardial injury [65,66].	ANCA-associated neutrophil activation, eosinophil cytotoxic proteins, complement activation, and inflammatory mediators [11,36,65,66].	Small-vessel vasculitis leads to myocardial ischemia, necrosis, and inflammatory remodeling [1,58,65,66].	Myocarditis, ventricular dysfunction, arrhythmias, heart failure syndromes [65,66].	CMR identifies myocardial inflammation; eosinophilia and inflammatory markers support diagnosis. Rapid immunosuppressive therapy is essential [65,66].

Abbreviations: ANCA—anti-neutrophil cytoplasmic antibodies; CMR—cardiac magnetic resonance; EGPA—eosinophilic granulomatosis with polyangiitis; IFN—interferon; IIM—idiopathic inflammatory myopathies; IL—interleukin; NF-κB—nuclear factor kappa B; RA—rheumatoid arthritis; SLE—systemic lupus erythematosus; SSc—systemic sclerosis; TNF—tumor necrosis factor.

## Data Availability

No new data were created or analyzed in this study. Data sharing is not applicable to this article.

## Abbreviations

ADMA	asymmetric dimethylarginine
AMPK	AMP-activated protein kinase
ANCA	anti-neutrophil cytoplasmic antibodies
APS	antiphospholipid syndrome
ATF6	activating transcription factor 6
BH4	tetrahydrobiopterin
BNP	B-type natriuretic peptide
CFR	coronary flow reserve
CMD	coronary microvascular dysfunction
CMR	cardiac magnetic resonance
CRP	C-reactive protein
DAMPs	damage-associated molecular patterns
ECM	extracellular matrix
ECV	extracellular volume
EGPA	eosinophilic granulomatosis with polyangiitis
eNOS	endothelial nitric oxide synthase
ER	endoplasmic reticulum
ESR	erythrocyte sedimentation rate
GDF-15	growth differentiation factor-15
GPX4	glutathione peroxidase 4
HIF-1α	hypoxia-inducible factor-1α
hs-cTn	high-sensitivity cardiac troponin
IFN	interferon
IIM	idiopathic inflammatory myopathies
IL	interleukin
IRE1α	inositol-requiring enzyme 1α
JAK	Janus kinase
KLF	Krueppel-like factor
mTOR	mechanistic target of rapamycin
mtDNA	mitochondrial DNA
mtROS	mitochondrial reactive oxygen species
NETs	neutrophil extracellular traps
NF-κB	nuclear factor kappa B
NLR	NOD-like receptor
NLRP3	NOD-like receptor family pyrin domain containing 3
NO	nitric oxide
Nrf2	nuclear factor erythroid 2-related factor 2
NT-proBNP	N-terminal pro-B-type natriuretic peptide
PAR	protease-activated receptor
PERK	protein kinase RNA-like ER kinase
PET	positron emission tomography
PKM2	pyruvate kinase M2
RA	rheumatoid arthritis
RIPK	receptor-interacting protein kinase
ROS	reactive oxygen species
SANRA	Scale for the Assessment of Narrative Review Articles
SLE	systemic lupus erythematosus
sST2	soluble suppression of tumorigenicity-2
SSc	systemic sclerosis
TGF-β	transforming growth factor-β
Th	T helper
TLR	Toll-like receptor
TNF	tumor necrosis factor
